# Neuroprotective Actions of Different Exogenous Nucleotides in H_2_O_2_-Induced Cell Death in PC-12 Cells

**DOI:** 10.3390/molecules28031226

**Published:** 2023-01-26

**Authors:** Na Zhu, Riu Liu, Mei-Hong Xu, Yong Li

**Affiliations:** 1 Department of Nutrition and Food Hygiene, College of Public Health, Inner Mongolia Medical University, Hohhot 010059, China; 2 Department of Nutrition and Food Hygiene, School of Public Health, Peking University, Beijing 100191, China; 3 Beijing Key Laboratory of Toxicological Research and Risk Assessment for Food Safety, Peking University, Beijing 100191, China

**Keywords:** exogenous nucleotides, neuroprotective, neurodegenerative, oxidative stress, inflammation, mitochondrial function

## Abstract

Exogenous nucleotides (NTs) are considered conditionally essential nutrients, and the brain cannot synthesize NTs de novo. Therefore, the external supplementation of exogenous NTs is of great significance for maintaining normal neuronal metabolism and function under certain conditions, such as brain aging. This study, therefore, sets out to assess the neuroprotective effect of four kinds of single exogenous NTs and a mixture of the NTs, and to elucidate the potential mechanism. A rat pheochromocytoma cell line PC-12 was treated with different concentrations of exogenous NTs after 4 h of exposure to 200 µM H_2_O_2_. We found that the exogenous NTs exerted significant neuroprotection through decreasing neuron apoptosis and DNA damage, ameliorating inflammation and mitochondrial dysfunction, promoting cell viability, and augmenting antioxidant activity, and that they tended to up-regulate the NAD^+^/SIRTI/PGC-1α pathway involved in mitochondrial biogenesis. Among the different NTs, the neuroprotective effect of AMP seemed to be more prominent, followed by the NT mixture, NMN, and CMP. AMP also exhibited the strongest antioxidant activity in H_2_O_2_-treated PC-12 cells. UMP was excellent at inhibiting neuronal inflammation and improving mitochondrial function, while GMP offered major advantages in stabilizing mitochondrial membrane potential. The mixture of NTs had a slightly better performance than NMN, especially in up-modulating the NAD^+^/SIRTI/PGC-1α pathway, which regulates mitochondrial biogenesis. These results suggest that antioxidant activity, anti-inflammatory activity, and protection of mitochondrial function are possible mechanisms of the neuroprotective actions of exogenous NTs, and that the optimization of the mixture ratio and the concentration of NTs may achieve a better outcome.

## 1. Introduction

Increased life expectancies have greatly expanded the number of elderly individuals with neurodegenerative disorders, such as Parkinson’s disease (PD) and Alzheimer’s disease (AD), as the major risk factor for developing these conditions is age. Currently, more than 55 million people suffer from neurodegenerative disorders worldwide. The financial cost of treating and caring for those with neurodegenerative disorders exceeds the expense of treating individuals with cancer and cardiovascular disease combined, which inflicts a heavy economic burden on society [1]. Thus, efforts to facilitate successful brain aging are worthwhile.

Neurodegenerative disorders are characterized by the deposition of neurotoxic proteins resulting from dysfunction and degeneration of specific populations of neurons in the brain, which experience oxidative and metabolic stress during the aging process. The hallmarks of the neural dysfunction or degeneration include mitochondrial dysfunction [2], accumulation of oxidatively damaged molecules [3], dysregulated energy metabolism [4], autophagy dysfunction [5], impaired DNA repair [6], disruption of neuronal networks [7], altered calcium homeostasis [8], and inflammation [9]. Given these facts, reducing the risk of neurodegenerative diseases requires eliminating neurotoxic proteins and improving neuronal function.

There are a growing number of studies that have demonstrated that dietary interventions, including dietary restriction, could modify brain aging and the development of neurodegenerative disorders. Mild cellular stress induced by dietary restriction up-modulates the expression of the coding proteins of genes, which are essential to neuron survival, and it may be especially successful at lowering the risk of age-associated neurodegenerative disorders [10]. Dietary factors such as vitamin E [11] and creatine [12] possess antioxidant properties, and can shield neurons from a range of oxidative assaults during brain aging and the development of neurodegenerative diseases. Dietary restriction and dietary supplementation of coenzyme Q10 [13] and naringenin [14] can stabilize mitochondrial function to counteract the energy deficits that occur during neural dysfunction. Dietary interventions, such as the supplementation of curcumin and ginsenosides [15,16], perform neuroprotective roles in neurodegenerative models via anti-inflammatory activities. In addition, dietary restriction and melatonin supplementation can inhibit the accumulation of neurotoxic proteins through increasing the autophagy pathway [17,18].

NTs are composed of pentose, base, and phosphoric acid, they are the fundamental components of nucleic acid. NTs are involved in determining the characteristics of organisms and the structure and function of a protein, orchestrating a myriad of physiological and biochemical functions in biological organisms, and participating in the regulation of various substances of metabolism. NTs serve as the primary high-energy chemicals in the pathway for energy metabolism and crucial messengers in the transmission of cell signals. NTs can be synthesized endogenously or can be ingested exogenously through dietary intake. Extensive research has confirmed the health benefits of exogenous NTs, including antioxidant activity, immunomodulatory activity, DNA protective activity, the promotion of cell proliferation, the maintenance of liver and intestinal function [19], and the restoration of mitochondrial function [20]. These bioactive traits point to the possible contribution of NTs to lowering the risk of neurodegenerative disorders. NTs cannot be synthesized de novo by the brain and bone marrow, and the salvage pathway is the principal alternative for the synthesis of NTs in the brain. Therefore, we assume that the external supplementation of NTs is of great significance for neuronal dysfunction and energy deficiency during brain aging. Moreover, evidence from several studies suggests that NTs may enhance brain nutrition and function [19], and inhibit age-induced deterioration of brain morphology and memory [21]. In addition, recent work performed in our laboratory revealed that exogenous NTs are involved in the synthesis of NAD^+^, consequently leading to the promotion of mitochondrial biogenesis via the NAD^+^/ SIRT1/PGC-1α pathway in the H_2_O_2_-induced senescent NIH/3T3 cells model. Collectively, all of these facts drove us to assess the significance of NTs to neural dysfunction during brain aging or the development of neurodegenerative disorders. This work examines the neuroprotective function of exogenous NTs and demonstrates the distinct roles of four different types of single NTs and a mixture of the NTs using an H_2_O_2_-induced neurodegenerative PC-12 cell model.

## 2. Results

### 2.1. NTs Protect against Neurodegeneration of H_2_O_2_-Treated PC-12 Cells

**Figure 1:**
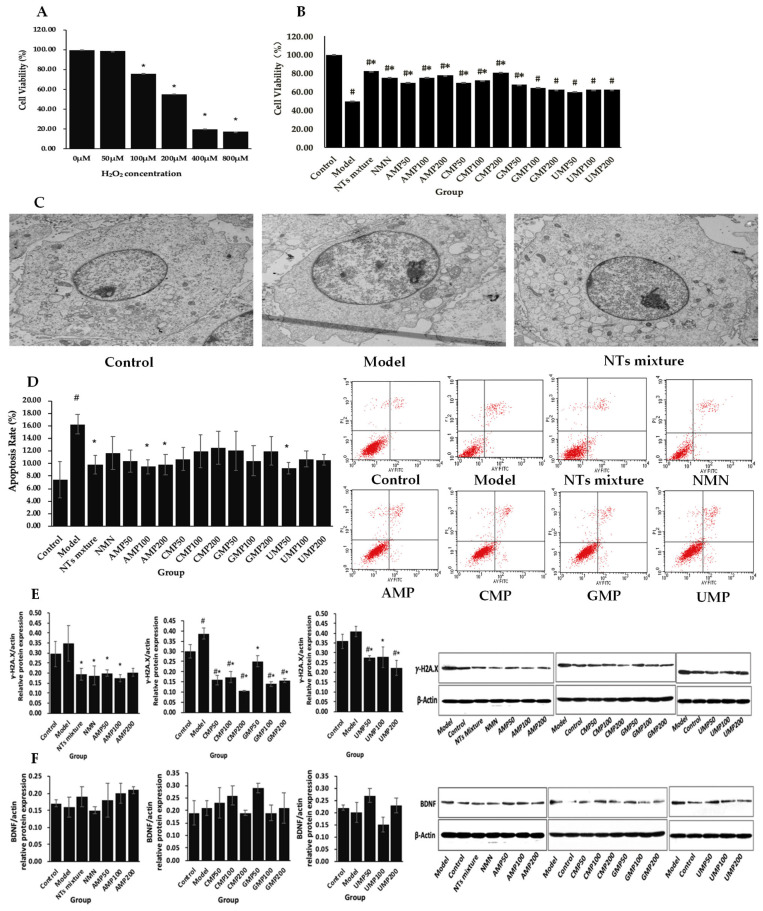
(**A**) The dose-dependent response of H_2_O_2_ to PC-12 cells; (**B**) The cell viability evaluation of NTs using the CCK-8 assay; (**C**) The effect of NTs on the morphological changes of H_2_O_2_-treated PC-12 cells using transmission electron microscopy (3000×); (**D**) The effect of NTs on the cell apoptosis rate in H_2_O_2_-treated PC-12 cells; (**E**) The effect of NTs on the protein expression of γ-H2A.X in H_2_O_2_-treated PC-12 cells; (**F**) the effect of NTs on the protein expression of BDNF in H_2_O_2_-treated PC-12 cells (*n* = 3 per group). # *p* < 0.05 versus control group, * *p* < 0.05 versus model group.

The preliminary test demonstrated that 4 h of H_2_O_2_ treatment significantly decreased cell viability in the range of 100~800 µM, in which 200 µM H_2_O_2_ decreased the cell viability to 55% (Figure 1A). In the follow-up study, PC-12 was treated with 200 µM H_2_O_2_ for 4 h. A supplementation of the NT mixture, NMN, AMP50/100/200, CMP50/100/200, and GMP50 significantly increased the cell viability of H_2_O_2_-treated PC-12 cells compared with the model group (Figure 1B). The increased rates relative to the model group were 64.44% (mixture of NTs) > 62.22% (CMP200) > 55.56% (AMP200) > 51.11% (NMN/AMP100) > 44.44% (CMP100) > 40.00% (AMP50/CMP50) > 35.56% (GMP50).

H_2_O_2_-mediated neurotoxic effects changed the morphological structure of PC-12, and led to the display of an enlarged nucleus, chromatin pyknosis, irregular nuclear membranes, reduced mitochondrial numbers, and increased mitochondrial size. Compared with the model group, the PC-12 cells supplemented with the 100 µM NT mixture displayed normal nucleus sizes, homogeneous chromatin, flat nuclear membranes, and relatively increased numbers of mitochondria of normal sizes (Figure 1C).

Increased cell apoptosis was witnessed in H_2_O_2_-treated PC-12 cells. The supplementation of the NT mixture, AMP100/200, and UMP50 significantly decreased the cell apoptosis rate in the H_2_O_2_-treated PC-12 cells (Figure 1D). The decreased rates relative to the model group were 43.17% (UMP50) > 41.30% (AMP100) > 39.37% (AMP200) >39.27% (NT mixture).

Oxidative stress frequently targets DNA during healthy metabolism, aging, and other stress responses. We found that the protein expression of γ-H2A.X, a marker of DNA damage, up-regulated in H_2_O_2_-treated PC-12 cells (Figure 1E). The supplementation of four kinds of single NTs, a mixture of the NTs, and NMN significantly down-regulated the protein expression of γ-H2A.X compared with the model group; only the AMP200 failed to suppress its expression. The decreased rates relative to the model group were 72.84% (CMP200) > 64.15% (GMP100) > 59.76% (GMP200) > 59.25% (CMP50) > 55.30% (CMP100) > 49.58% (AMP100) > 46.22% (NMN) > 45.75% (UMP200) > 44.49% (NT mixture) > 42.57% (AMP50) > 35.18% (GMP50) > 33.03% (UMP50) > 31.81% (UMP100).

The expression of the brain-derived neurotrophic factor (BDNF) did not show a significant change in the present cell model. Compared with the model group, the supplementation of four kinds of single NTs, the NT mixture, and NMN had no significant influence on the level of this protein (Figure 1F).

### 2.2. NTs Suppress Neuroinflammation in H_2_O_2_-Treated PC-12 Cells

**Figure 2:**
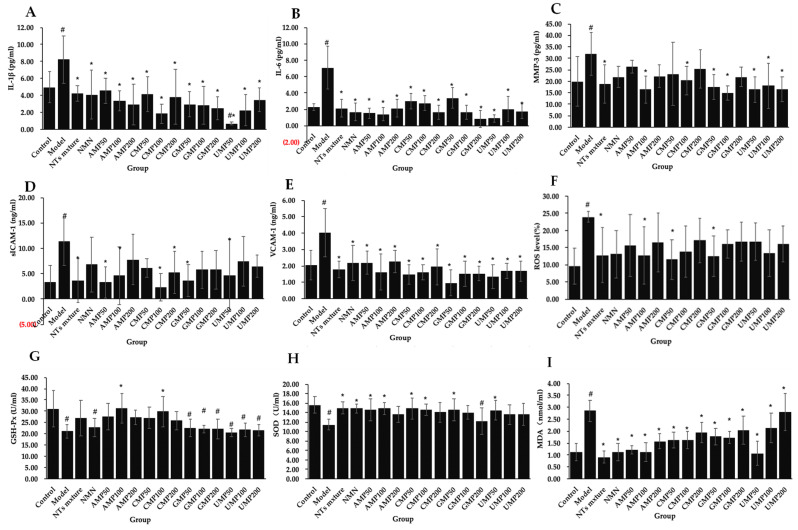
(**A**) The effect of NTs on the secretion of IL-1𝛽 in H_2_O_2_-treated PC-12 cells; (**B**) The effect of NTs on the secretion of IL-6 in H_2_O_2_-treated PC-12 cells; (**C**) The effect of NTs on the secretion of MMP3 in H_2_O_2_-treated PC-12 cells; (**D**) The effect of NTs on the secretion of sICAM-1 in H_2_O_2_-treated PC-12 cells; (**E**) The effect of NTs on the secretion of VCAM-1 in H_2_O_2_-treated PC-12 cells; (**F**) The effect of NTs on the intracellular ROS production in H_2_O_2_-treated PC-12 cells; (**G**) The effect of NTs on the GSH-Px activities in H_2_O_2_-treated PC-12 cells; (**H**) The effect of NTs on the SOD activities in H_2_O_2_-treated PC-12 cells; (**I**) The effect of NTs on the MDA levels in H_2_O_2_-treated PC-12 cells (*n* = 3 per group). # *p* < 0.05 versus control group, * *p* < 0.05 versus model group.

H_2_O_2_ exposure caused neuroinflammation, a common feature of brain aging, in PC-12 cells. An increased secretion of IL-6, IL-1β, MMP-3, sICAM-1, and VCAM-1 was observed in H_2_O_2_-treated PC-12 cells compared with the control group. The supplementation of four kinds of single NTs, the NT mixture, and NMN all significantly decreased IL-6 (Figure 2A), IL-1β (Figure 2B), and VCAM-1 secretion (Figure 2E), all at a decreased rate of up to 44.71%. The NT mixture, AMP100, CMP100, GMP50/100, and UMP50/100/200 significantly decreased MMP-3 concentration (Figure 2C) compared with the model group. The decreased rates were 53.02% (GMP100) > 48.70% (AMP100) > 48.59% (UMP50) > 48.43% (UMP200) > 44.65% (GMP50) > 43.50% (UMP100) > 41.19 (NT mixture) > 36.40% (CMP100). The NT mixture, AMP50/100, CMP100/200, GMP50, and UMP50 significantly decreased the sICAM-1 level (Figure 2D) compared with the model group. The decreased rates were 79.09% (CMP100) > 70.71% (AMP50) > 68.17% (NT mixture) > 67.90% (GMP50) > 59.14% (AMP100) > 58.93% (UMP50) > 53.75% (CMP200).

### 2.3. NTs Inhibit ROS Production and Enhance Antioxidant Activities in H_2_O_2_-Treated PC-12 Cells

The H_2_O_2_-treated PC-12 cells generated significantly higher levels of ROS compared with the control group. Significantly decreased ROS production was seen in the PC-12 cells supplemented with the NT mixture, AMP100, CMP50 and GMP50 (Figure 2F). The decreased rates were 51.98% (CMP50) > 47.81% (GMP50) > 46.73% (AMP100) > 46.69% (NT mixture). We also found that AMP100 and CMP100 significantly enhanced the GSH-Px activities compared with the model group (Figure 2G). The increased rates were 47.62% (AMP100) and > 40.95% (CMP100). The supplementation of the NT mixture, NMN, AMP50/100, CMP50/100, GMP50 and UMP50 significantly enhanced SOD activities in PC-12 cells (Figure 2H). The increased rates were 31.00% (NT mixture) > 29.86% (NMN) > 29.67% (AMP100/CMP50) > 27.75% (AMP50) > 27.56% (GMP50) > 26.99% (CMP100) > 26.79% (UMP50). In addition, the supplementation of four kinds of single NTs, the NT mixture, and NMN all significantly reduced the MDA production in H_2_O_2_-treated PC-12 cells (Figure 2I); the highest inhibition rate was 67.86% (NT mixture).

### 2.4. NTs Improve Mitochondrial Function and Tend to Up-Regulate the Mitochondrial Biogenesis Related Pathway NAD^+^/SIRT1/PGC-1α in H_2_O_2_-Treated PC-12 Cells

**Figure 3:**
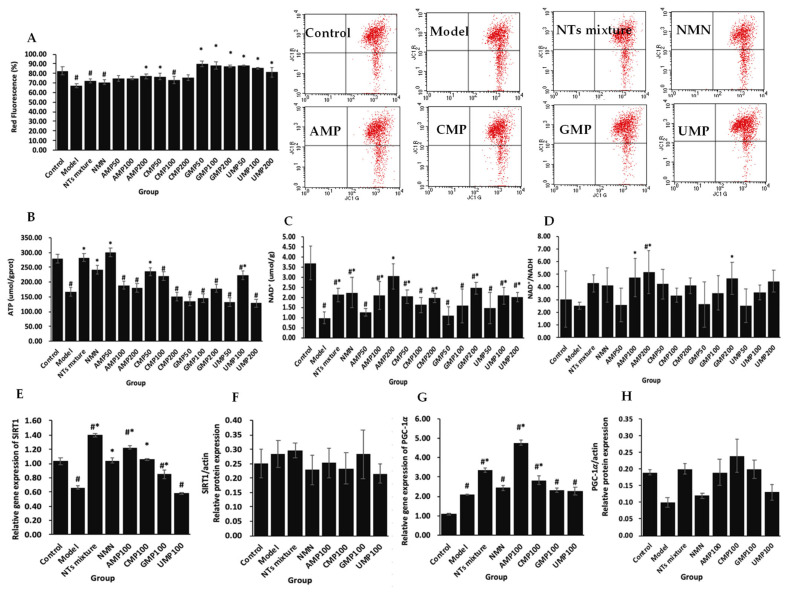
(**A**) The effect of NTs on the mitochondrial membrane potential in H_2_O_2_-treated PC-12 cells; (**B**) The effect of NTs on the ATP production in H_2_O_2_-treated PC-12 cells; (**C**) The effect of NTs on the NAD^+^ levels in H_2_O_2_-treated PC-12 cells; (**D**) The effect of NTs on NAD^+^/NADH in H_2_O_2_-treated PC-12 cells; (**E**) The effect of NTs on the relative gene expression of SIRT1 in H_2_O_2_-treated PC-12 cells; (**F**) The effect of NTs on the relative protein expression of SIRT1 in H_2_O_2_-treated PC-12 cells; (**G**) The effect of NTs on the relative gene expression of PGC-1α in H_2_O_2_-treated PC-12 cells; (**H**) The effect of NTs on the relative protein expression of PGC-1α in H_2_O_2_-treated PC-12 cells. Values represented the mean ± S.D. (*n* = 3 per group). # *p* < 0.05 versus control group, * *p* < 0.05 versus model group.

Mitochondrial dysfunction is characterized by the depolarization of mitochondrial membrane potential and the decrement of ATP production in H_2_O_2_-treated PC-12 cells compared with the control group. AMP200, CMP50 and different concentrations of GMP and UMP supplementation significantly increased the rate of red fluorescent relative to the model group (Figure 3A). The increased rates were 33.15% (GMP50) > 31.06% (UMP50) > 31.02% (GMP100) > 29.96% (GMP200) > 26.86 (UMP100) > 20.76% (UMP200) > 14.15% (AMP200) > 13.86% (CMP50). The supplementation of the NT mixture, NMN, AMP50, CMP50, and UMP100 significantly increased ATP production compared with the model group (Figure 3B). The increased rates were 79.59% (AMP50) > 68.56% (NT mixture) > 44.24% (NMN) > 40.24% (CMP50) > 33.15% (UMP100).

Further analysis showed that NTs may promote mitochondrial biogenesis through increasing NAD^+^ activity and up-regulating the gene and protein expression of SIRT1 and PGC-1α. Compared with the control group, H_2_O_2_ treatment significantly decreased NAD^+^ levels, and tended to lower NAD^+^/NADH. The supplementation of the NT mixture, NMN, AMP100/200, CMP50/200, GMP200, and UMP100/200 significantly increased NAD^+^ levels (Figure 3C). The increased rates were 208.98% (AMP200) > 149.46% (GMP200) > 127.48% (NMN) > 115.38% (NT mixture) > 111.86% (AMP100) > 111.07% (UMP100) > 106.56% (CMP50) > 105.02% (UMP200) > 100.15% (CMP200). AMP100/200 and GMP200 supplementation also significantly increased NAD^+^/NADH (Figure 3D). The increased rates were 104.47% (AMP200) > 87.97% (AMP100), and 85.13% (GMP200). H_2_O_2_ exposure significantly down-modulated the gene expression of SIRT1 in PC-12 cells, and the supplementation of the NT mixture, NMN, AMP, CMP, and GMP reversed this trend completely (Figure 3E). The increased rates were 110.18% (NT mixture) > 84.32% (AMP100) > 59.07% (CMP100) > 56.17% (NMN) > 28.02% (GMP100). The protein expression of SIRT1 tended to increase in the model group. Compared with the model group, the NT mixture supplementation tended to increase the protein expression of SIRT1 (Figure 3F). All of the above results suggest that decreased NAD^+^ activity results in decreased consumption of the NAD^+^ -dependent protein, SIRT1, in the model group. The administration of NTs significantly increased the NAD^+^ activity and gene expression of SIRT1, and maintained the same expression level of SIRT1 protein as that of the model group.

Interestingly, oxidative stress was found to up-regulate the gene expression of PGC-1α in PC-12 cells, while the supplementation of the NT mixture, AMP, and CMP further increased the gene expression of PGC-1α (Figure 3G). The increased rates were 127.33% (AMP100) > 58.99% (NT mixture) > 34.96% (CMP100). Compared with the model group, the protein expression of PGC-1α tended to increase in the groups administered with NTs (Figure 3H).

## 3. Discussion

NTs are the fundamental units of nucleic acid macromolecules, which are involved in different physiological and regulatory functions in organisms. Exogenous NTs were obtained from enzymatic hydrolysis of nucleic acid-enriched food. As basic raw materials, exogenous supplementation of NTs under specific physiological conditions can exert multiple effects in various biological systems, and all the bioactive properties confer the neuroprotective potential of NTs. The initial objective of the present project was to investigate the neuroprotective effect of exogenous NTs in H_2_O_2_-treated PC-12 cells and validate the underlying mechanism. We showed that exogenous NT supplementation protects against the neurodegeneration of H_2_O_2_-treated PC-12 cells, as demonstrated by increased cell viability, decreased inflammation, apoptotic rate, DNA damage, and morphology changes. Our findings accord with earlier observations that salmon milt and yeast-derived nucleic acid enhance the resistance of cells to oxidative stress, and inhibit apoptosis in A𝛽-treated SH-SY5Y neuronal cells [22]. Exogenous NTs also provide significant neuroprotection in in vivo models of nervous system diseases. Chen et al. found that the exogenous supplementation of a nucleoside-nucleotide mixture was associated with decreased age-induced deterioration of brain morphology and certain memory tasks [21]. Sato et al. found that a diet supplemented with NTs raised the levels of n-3 and n-6 lipids in the brain and enhanced the learning ability of rats [23]. Our findings are in line with those of previous studies. In addition, our results further support the idea that NTs reduce DNA damage [24,25] and cell apoptosis [26,27], and promote cell proliferation [28,29].

We hypothesize that one possible mechanism of neuroprotective action of exogenous NTs is their antioxidant activity. In this study, exogenous NTs were found to reduce ROS and MDA production, and enhance GSH-Px and SOD activities in H_2_O_2_-treated PC-12 cells. Previous in vivo experiments in our laboratory also indicated the antioxidant characteristics of NTs in different tissues and systems [30,31]. This study was the first to show the antioxidative action of exogenous NTs on neurons. NTs are non-enzymatic antioxidants, and as the raw materials for the synthesis of nucleic acids, NTs involved in amino acid metabolism may in turn influence the expression, activities, or metabolism of antioxidant enzymes such as glutathione. NTs play a crucial regulatory role in the synthesis of unsaturated fatty acids. Several reports have shown that NTs added to infant formulas play a crucial role in the desaturation of essential fatty acids and the extension of long-chain polyunsaturated fatty acids in infants [32,33]. Moreover, exogenous NT supplementation was effective in reversing lipid metabolism abnormalities in a rat model of liver cirrhosis [34], while unsaturated fatty acids were able to neutralize ROS and enhance the expression of the antioxidant gene in neurons [35]. Thus, NTs diminished oxidative stress in neurons, via direct or indirect actions, thereby reducing the accumulation of oxidatively damaged macromolecules, down-regulating the stress-activated signaling pathway, and enabling the homeostasis of neurons.

Neuroinflammation is known to contribute to the neuronal damage that underpins neurodegenerative disorders [36]. In addition, neuroinflammation is closely connected with the blood-brain barrier disruption, thus destroying neuronal energy homeostasis. We found that the administration of exogenous NTs significantly inhibited neuroinflammation in H_2_O_2_-treated PC-12 cells, demonstrating that an anti-inflammatory mechanism may be responsible for the neuroprotective effects of exogenous NTs. In accordance with the present results, numerous earlier investigations have shown that exogenous NTs have potent immune-boosting and inflammation-controlling effects. Researchers found that the content of NTs in formula milk was lower than that in breast milk, which led to low immunity in infants. When exogenous NTs were added to formula milk, the incidence of acute diarrhea in infants was significantly decreased [37]. In another study, exogenous NT feed was able reduce the levels of IL-1 and IFN-γ and increase the level of IL-10 in an inflammatory reaction mice model, thus regulating the balance of inflammatory/anti-inflammatory cytokines in mice [38]. The anti-neuroinflammation action of exogenous NTs has not been studied. The present study was the first to demonstrate the anti-neuroinflammatory effects of exogenous NTs, and revealed that UMP was the most effective at inhibiting the secretion of inflammatory cytokines in H_2_O_2_-treated PC-12 cells.

Mitochondria are distributed in neurons and produce ATP to support the normal physiological activity of neurons. Numerous investigations have revealed age-related mitochondrial alterations, including mitochondrial enlargement, depolarized membrane potential, and decreased ATP production [39,40]. Consistent with previous findings, in the present study, the age-related mitochondrial alterations mentioned above were observed in H_2_O_2_-treated PC-12 cells, while exogenous NT treatment significantly increased mitochondrial membrane potential, ATP production, and normalized mitochondria size. These results are in accord with other studies, indicating that exogenous NTs stimulate the generation and storage of intracellular energy, promote mitochondrial DNA repair, and enhance mitochondrial enzyme activities [20,41,42]. Mitochondrial dysfunction during brain aging is also associated with a decrement in cellular NAD^+^ levels and NAD^+^/NADH. Our findings provide more evidence of this link, and exogenous NT supplementation reversed this trend. Moreover, the effect of AMP200 on increasing NAD^+^ levels was superior to NMN. On the other hand, as mentioned in the introduction, our recent work shows that exogenous NTs up-regulate the NAD^+^/SIRT1/PGC-1α pathway, which can regulate the biogenesis and electron transport systems of mitochondria [43] in senescent mouse embryonic fibroblasts. Similarly, the present study showed a positive association between NT supplementation and the up-modulation of the gene and protein expression of SIRT1 and PGC-1α. The above results imply a neuroprotective role of exogenous NTs against H_2_O_2_ exposure through the promotion of mitochondrial biogenesis via the NAD^+^/SIRT1/PGC-1α signaling pathway. In addition, researchers have found that SIRT1 is neuroprotective in AD models through the regulation of Aβ metabolism, and that its deletion causes increased tau acetylation, phosphorylation, and cognitive defects [44]. Increased neuronal SIRT1 expression might be another possible mechanism contributing to the neuroprotective performance of exogenous NTs.

We are dedicated to the universal concept that exogenous NTs are essential nutrients required for age-specific stages in life. The present study has been one of the first attempts to extensively examine the effect of exogenous NTs, including four kinds of single NTs and a mixture of the NTs, on H_2_O_2_-induced neural dysfunction. NMN is a derivative of NTs and shares a similar chemical structure, and therefore served as a positive control in the present study. Numerous studies have demonstrated that NMN also provides neuroprotection as NAD^+^ precursors [45,46]. Overall, the neuroprotective effect of AMP seemed to be more prominent, followed by the NT mixture, NMN, and CMP. When it came to increasing antioxidant activity and mitochondrial biogenesis, they all performed better. Different exogenous NTs, the NT mixture, and NMN all significantly inhibited the neuronal inflammatory response. Among them, UMP was the most effective. UMP was also superior in improving mitochondrial function, and only UMP100 was able to maintain stable levels of ATP and mitochondrial membrane potential, while GMP offered major advantages in stabilizing mitochondrial membrane potential. These results suggest that the optimization of the NT mixture ratio and concentration may achieve a better outcome. NTs performed slightly better than NMN, especially in up-modulating the mitochondrial biogenesis-related gene expression of SIRT1 and PGC-1α. Furthermore, as nutrients, the safety of NTs is much higher than that of NMN. In addition, we speculated that the protective effect of exogenous NTs on the nervous system is multifold. Our previous study found that exogenous NTs delayed endothelial cell senescence [47], implying that they may have a certain protective effect on the blood-brain barrier, which serves two roles: supplying nutrients and blocking inflammatory insults [48]. Large-scale studies showed that the blood-brain barrier is broken down by various neurodegenerative diseases [49]. Therefore, exogenous NTs may retain the homeostasis of the nervous system by maintaining the integrity of the blood-brain barrier. Unquestionably, more work will be needed to confirm our assumption.

There were several limitations in our study. First, we only verified the signaling pathway that correlated with previous findings, without in-depth mechanism research. Further multi-omics studies should be performed to investigate a wider array of signaling pathways with a full consideration of the histiocytic specificity. Second, the present study simulated the model of neuronal dysfunction, which is the pathological basis of neurodegenerative diseases, making our findings less generalizable to protein-aggregative neurodegenerative diseases. Therefore, more sophisticated cellular and animal models are needed to explore the effects of exogenous NTs on protein-aggregative neurodegenerative diseases such as AD and PD.

## 4. Materials and Methods

### 4.1. Chemicals

The NTs used in our experiment were extracted from ribonucleic acid by enzymatic hydrolysis, including disodium guanosine-5′-monophosphate (GMP), disodium 5′- uridine monophosphate (UMP), 5′-cytimidine monophosphate (CMP), 5′-adenosine monophosphate (AMP), and nicotinamide mononucleotide (NMN), respectively. All of them were supplied by Dalian ZHEN-AO Biotechnology Co. Ltd. (Dalian, China).

### 4.2. Cell Cuture and Teatments

PC-12 were obtained from American Type Culture Collection (ATCC, Manassas, VA, USA). The PC-12 were cultured in Dulbecco’s Modification of Eagle’s Medium (DMEM) (GIBCO, Grand Island, NE, USA), and supplemented with 10% fetal bovine serum (Zhong Qiao Xin Zhou Biotechnology Co. Ltd., Shanghai, China) and 1% antibiotic–antimitotic (Coolaber, Beijing, China) at 37 °C under a humidified atmosphere of 5% CO_2_.

H_2_O_2_-induced cell injury was performed as previously described; the PC-12 were maintained for 4 h in a growth medium containing different concentrations of H_2_O_2_, and then cultured in the growth medium. Cell viability was assessed after 24 h and the appropriate H_2_O_2_ concentration was selected for the following experiments.

With the exception of the control group (growth medium without H_2_O_2_) and the model group (growth medium with 200µM H_2_O_2_), the cells (exposed to H_2_O_2_ for 4 h) were treated with different concentrations of single NTs, the NT mixture, and NMN for 24 h, as follows: the NT mixture group (growth medium containing a 100 µM NT mixture; AMP: CMP: GMP: UMP = 22.80: 25.80: 30.20: 20.40), the NMN group (growth medium containing 0.5 mM NMN), and low, middle, and high doses of GMP/UMP/CMP/AMP groups (growth medium containing 50/100/200 µM GMP/UMP/CMP/AMP). Following exposure to the NTs, the cells were harvested for further analysis.

### 4.3. Morphology Observation

The cell morphological changes were observed by a transmission electron microscope. In brief, cells were collected and washed twice with PBS before being fixed with 2.5% glutaraldehyde overnight at 4 °C. Then, cells were washed three times with PBS and fixed with 1% osmic acid for 1 h. Graded acetone was dehydrated (30%, 70%, 95%, and 100% in PBS, each for 15 min), the resin was embedded, and ultrathin sections (Mode: OMU3, Leica Reichert, Munich, Germany) were stained with uranyl acetate and citric acid (Beyotime, Shanghai, China). Cellular morphology and mitochondria were observed by transmission electron microscopy (JEM-1400. Leica Reichert, Wetzlar, Germany).

### 4.4. Cell Viability Assay

Cell viability was evaluated by the cell-counting kit-8 (CCK-8) assay (KeyGEN, Jiangsu, China) according to the manufacturer’s protocol. In brief, 100 µL/well cells (about 1 × 10^4^) were seeded in 96-well plates. After treatment according to the protocol, 10 µL CCK-8 was added to each well and incubated at 37 °C for 1–4 h. The absorbance of each well was measured at 450 nm with a microplate reader (BMG FLUOstar Omega, Offenburg, Germany).

### 4.5. Flow Cytometry

First, 2 mL/well cells (about 2 × 10^5^) were seeded in 6-well plates and treated according to the protocol. For apoptosis analysis, cells were harvested and washed once with PBS and then resuspended in PI/Annexin-V solution (KeyGEN, Nanjing, China) and analyzed using a Flow Cytometer (Beckman Coulter, Brea, CA, USA). For intracellular ROS analysis, cells were harvested and washed once with PBS before being incubated for 20 min at 37 °C with a 10 µM 2,7-dichlorofluorescein diacetate (Beyotime, Shanghai, China). After being washed with PBS three times, the cells were analyzed using a Flow Cytometer (Beckman Coulter, Brea, CA, USA). For mitochondrial membrane potential (∆Ψm) analysis, cells were harvested and stained with a 500 µL 1× JC-1 dye solution (Beyotime, Shanghai, China) at 37 °C for 20 min in the dark. Then, the cells were washed twice and resuspended using a 1× JC-1 staining buffer. The change of fluorescence color was analyzed using flow cytometry (Beckman Coulter, Brea, CA, USA).

### 4.6. Biochemical Analysis

First, 2 mL/well cells (about 2 × 10^5^) were seeded in 6-well plates and treated according to the protocol. Then, the supernatant was obtained for the measurements of malondialdehyde (MDA), glutathione peroxidase (GSH-Px), superoxide dismutase (SOD) activities, NAD^+^/NADH, ATP, interleukin-6 (IL-6), IL-1β, matrix metalloproteinase-3 (MMP-3), intercellular cell adhesion molecule-1 (ICAM-1), and vascular cell adhesion molecule-1 (VCAM-1) using commercial kits.

### 4.7. Quantitative Real-Time PCR Analysis

The total RNA was extracted from cell samples using a Trizol reagent (Invitrogen, Waltham, USA) according to standard protocols. An AM-MLV reverse transcriptase kit (Promega, Madison, Wisconsin, USA) was used to prepare cDNA. The levels of mRNA expression were quantified using a real-time PCR amplification kit and a ABI 7500 Real Time PCR System (Applied Biosystems, Carlsbad, CA, USA). The primers information used in the present study are presented in Table 1.

### 4.8. Western Blot Analysis

First, 2 mL/well cells (about 2 × 10^5^) were seeded in 6-well plates and treated according to the protocol. Cells were collected and washed twice with PBS; then, they were resuspended in RIPA Lysis Buffer (Biosharp, Hefei, China) and supplemented with 1 mM of phenylmethanesulfonyl fluoride. Protein was extracted by centrifugation at 14,000× *g* for 15 min at 4 °C, and the concentration of protein was measured with a BCA protein assay kit (Thermo Scientific, Waltham, USA). Equal amounts of protein (80–150 µg) were separated by 10–20% SDS-PAGE gel and transferred to PVDF membranes at different electric currents according to the size of protein molecules. The membranes were blocked for 2 h in 5% non-fat milk dissolved with Tris-buffered saline containing 0.05% Tween-20 (TBST) at room temperature. Protein expression was detected using a primary antibody γ-H2A.X (1:1000, Abcam, Cambridge, MA, USA), BDNF (1:5000, Abcam, Cambridge, MA, USA), AMPKα (1:1000, CST, Danvers, MA, USA), PGC-1α (1:2000, Abcam, Cambridge, MA, USA), ULK2 (1:1000, CST, Danvers, MA, USA), SIRT1 (1:1000, CST, Danvers, MA, USA), β-actin (1:5000, Abcam, Cambridge, MA, USA), and horseradish peroxidase-conjugated anti-rabbit secondary antibodies (1:10,000, Abcam, Cambridge, MA, USA). A Quantitative analysis of a western blot was performed using Image-Pro Plus software (Media Cybernetics, Rockville, MD, USA).

### 4.9. Statistical Analysis

Statistical analyses were performed using *SPSS* software version 24 (*SPSS* Inc., Chicago, IL, USA). Data were expressed as mean ± standard deviation (SD) and analyzed by a one-way analysis of variance (ANOVA) test; a test for the difference of parametric samples among groups of multiple comparisons of least significant difference (equal variances assumed) or Dunnett’s T3 test (equal variances not assumed) was used. *p* < 0.05 indicated a statistically significant difference.

## 5. Conclusions

The present study was designed to evaluate the effect of four kinds of single NTs and a mixture of the NTs on H_2_O_2_-induced neuron degeneration. We showed that exogenous NTs exerted significant neuroprotection through decreasing neuron apoptosis and DNA damage, ameliorating inflammation and mitochondrial dysfunction, promoting cell viability, and augmenting antioxidant activity. Moreover, we found that NTs tend to up-modulate the longevity regulating pathway NAD^+^/SIRT1/PGC-1α, although further work is required to confirm this pathway. The neuroprotective effect of AMP seemed to be more prominent, followed by the NT mixture, NMN, and CMP. All of the NTs performed better in terms of boosting antioxidant activities and up-modulating the mitochondrial biogenesis-related NAD^+^/SIRT1/PGC-1α pathway. UMP is the most effective at inhibiting neuronal inflammation and improving mitochondrial function, while GMP offers major advantages in stabilizing mitochondrial membrane potential. The exogenous NT mixture performed slightly better than NMN, especially in terms of up-modulating the relative gene expression of SIRT1 and PGC-1. These results suggest that the optimization of the ratio and concentration of the NT mixture may achieve a better outcome.

## Figures and Tables

**Table 1:** The primers information used for real-time PCR analysis.

Primer Sequences	Probes	Sizes of PCR Products
AMPKα Forward primer	5′-GGGTGAAGATCGGCCACTAC-3′	164bp
AMPKα Reverse primer	5′-CTCTCTGCGGATTTTCCCGA-3′	
PGC-1α Forward primer	5′-GACTGGCAGGGGCACATCT-3′	156bp
PGC-1α Reverse Primer	5′-TGGGATGACCGAAGTGCTT-3′	
SIRT1 Forward primer	5′-TATGCTCGCCTTGCTGTAGA-3′	132bp
SIRT1 Reverse Primer	5′-TGGCTGGAATTGTCCAGGAT-3′	
ULK2 Forward primer	5′-TTAGTCAGTGCTGCTGTGGA-3′	99bp
ULK2 Reverse Primer	5′-AGAGTGACTGTGGTGACTGG-3′	
GAPDH Forward primer	5′-CAACTCCCTCAAGATTGTCAGCAA-3′	128bp
GAPDH Reverse primer	5′-GGCATGGACTGTGGTCATGA-3′	

## Data Availability

The data presented in this study are available on request from the corresponding author. The data are not publicly available due to privacy.

## References

[1] Hurd M.D., Martorell P., Langa K.M. (2013). Monetary costs of dementia in the United States. N. Engl. J. Med..

[2] Lin M.T., Beal M.F. (2006). Mitochondrial dysfunction and oxidative stress in neurodegenerative diseases. Nature.

[3] Simpson D.S.A., Oliver P.L. (2020). ROS Generation in Microglia: Understanding Oxidative Stress and Inflammation in Neurodegenerative Disease. Antioxidants.

[4] Mattson M.P., Arumugam T.V. (2018). Hallmarks of Brain Aging: Adaptive and Pathological Modification by Metabolic States. Cell Metab..

[5] Menzies F.M., Fleming A., Rubinsztein D.C. (2015). Compromised autophagy and neurodegenerative diseases. Nat. Rev. Neurosci..

[6] Chow H.M., Herrup K. (2015). Genomic integrity and the ageing brain. Nat. Rev. Neurosci..

[7] Mather M., Harley C.W. (2016). The Locus Coeruleus: Essential for Maintaining Cognitive Function and the Aging Brain. Trends Cogn. Sci..

[8] Strehler E.E., Thayer S.A. (2018). Evidence for a role of plasma membrane calcium pumps in neurodegenerative disease: Recent developments. Neurosci. Lett..

[9] Stephenson J., Nutma E., van der Valk P., Amor S. (2018). Inflammation in CNS neurodegenerative diseases. Immunology.

[10] Mattson M.P., Chan S.L., Duan W. (2002). Modification of brain aging and neurodegenerative disorders by genes, diet, and behavior. Physiol. Rev..

[11] Grundman M. (2000). Vitamin E and Alzheimer disease: The basis for additional clinical trials. Am. J. Clin. Nutr..

[12] Bender A., Klopstock T. (2016). Creatine for neuroprotection in neurodegenerative disease: End of story?. Amino Acids.

[13] Yang X., Zhang Y., Xu H., Luo X., Yu J., Liu J., Chang R.C. (2016). Neuroprotection of Coenzyme Q10 in Neurodegenerative Diseases. Curr. Top. Med. Chem..

[14] Chandran A.M.K., Christina H., Das S., Mumbrekar K.D., Satish Rao B.S. (2019). Neuroprotective role of naringenin against methylmercury induced cognitive impairment and mitochondrial damage in a mouse model. Environ. Toxicol. Pharmacol..

[15] Huang L., Chen C., Zhang X., Li X., Chen Z., Yang C., Liang X., Zhu G., Xu Z. (2018). Neuroprotective Effect of Curcumin against Cerebral Ischemia-Reperfusion via Mediating Autophagy and Inflammation. J. Mol. Neurosci..

[16] Cheng Z., Zhang M., Ling C., Zhu Y., Ren H., Hong C., Qin J., Liu T., Wang J. (2019). Neuroprotective Effects of Ginsenosides against Cerebral Ischemia. Molecules.

[17] Vohra M., Lemieux G.A., Lin L., Ashrafi K. (2017). The beneficial effects of dietary restriction on learning are distinct from its effects on longevity and mediated by depletion of a neuroinhibitory metabolite. PLoS Biol..

[18] Luo F., Sandhu A.F., Rungratanawanich W., Williams G.E., Akbar M., Zhou S., Song B.J., Wang X. (2020). Melatonin and Autophagy in Aging-Related Neurodegenerative Diseases. Int. J. Mol. Sci..

[19] Ding T., Song G., Liu X., Xu M., Li Y. (2021). Nucleotides as optimal candidates for essential nutrients in living organisms: A review. J. Funct. Foods.

[20] Pérez M.J., Sánchez-Medina F., Torres M., Gil A., Suárez A. (2004). Dietary nucleotides enhance the liver redox state and protein synthesis in cirrhotic rats. J. Nutr..

[21] Chen T.H., Wang M.F., Liang Y.F., Komatsu T., Chan Y.C., Chung S.Y., Yamamoto S. (2000). A nucleoside-nucleotide mixture may reduce memory deterioration in old senescence-accelerated mice. J. Nutr..

[22] Lam P.Y., Chen N., Chiu P.Y., Leung H.Y., Ko K.M. (2012). Neuroprotection against oxidative injury by a nucleic acid-based health product (Squina DNA) through enhancing mitochondrial antioxidant status and functional capacity. J. Med. Food.

[23] Sato N., Murakami Y., Nakano T., Sugawara M., Kawakami H., Idota T., Nakajima I. (1995). Effects of dietary nucleotides on lipid metabolism and learning ability of rats. Biosci. Biotechnol. Biochem..

[24] Korb V., Tep K., Escriou V., Richard C., Scherman D., Cynober L., Chaumeil J., Dumortier G. (2008). Current data on ATP-containing liposomes and potential prospects to enhance cellular energy status for hepatic applications. Crit. Rev. Ther. Drug Carr. Syst..

[25] Wang L.F., Gong X., Le G.W., Shi Y.H. (2008). Dietary nucleotides protect thymocyte DNA from damage induced by cyclophosphamide in mice. J. Anim. Physiol. Anim. Nutr..

[26] Puchałowicz K., Tarnowski M., Tkacz M., Chlubek D., Kłos P., Dziedziejko V. (2020). Extracellular Adenine Nucleotides and Adenosine Modulate the Growth and Survival of THP-1 Leukemia Cells. Int. J. Mol. Sci..

[27] Li M., Lu Y., Li Y., Tong L., Gu X.C., Meng J., Zhu Y., Wu L., Feng M., Tian N. (2019). Transketolase Deficiency Protects the Liver from DNA Damage by Increasing Levels of Ribose 5-Phosphate and Nucleotides. Cancer Res..

[28] Jang K.B., Kim S.W. (2019). Supplemental effects of dietary nucleotides on intestinal health and growth performance of newly weaned pigs. J. Anim. Sci..

[29] Holen E., Bjørge O.A., Jonsson R. (2005). Dietary nucleotides and human immune cells. II. Modulation of PBMC growth and cytokine secretion. Nutrition.

[30] Xu M., Liang R., Li Y., Wang J. (2017). Anti-fatigue effects of dietary nucleotides in mice. Food Nutr. Res..

[31] Xu M., Liang R., Guo Q., Wang S., Zhao M., Zhang Z., Wang J., Li Y. (2013). Dietary nucleotides extend the life span in Sprague-Dawley rats. J. Nutr. Health Aging.

[32] Wang L., Liu J., Lv H., Zhang X., Shen L. (2015). Effects of Nucleotides Supplementation of Infant Formulas on Plasma and Erythrocyte Fatty Acid Composition: A Meta-Analysis. PLoS ONE.

[33] Gil A., Lozano E., De-Lucchi C., Maldonado J., Molina J.A., Pita M. (1988). Changes in the fatty acid profiles of plasma lipid fractions induced by dietary nucleotides in infants born at term. Eur. J. Clin. Nutr..

[34] Leite L.H., Moreira-Vaz E., Rosa G., Pereira A.C., Monteiro C.R., Medeiros F.J., Chagas V.L. (2000). The influence of dietary nucleotides and long-chain polyunsaturated fatty acids on the incorporation of [3H] arachidonic acid on experimental liver cirrhosis. Arch. Latinoam. De Nutr..

[35] Al-Sabahi B.N., Fatope M.O., Essa M.M., Subash S., Al-Busafi S.N., Al-Kusaibi F.S., Manivasagam T. (2017). Pomegranate seed oil: Effect on 3-nitropropionic acid-induced neurotoxicity in PC12 cells and elucidation of unsaturated fatty acids composition. Nutr. Neurosci..

[36] Wang L.Y., Huang C.S., Chen Y.H., Chen C.C., Chen C.C., Chuang C.H. (2019). Anti-Inflammatory Effect of Erinacine C on NO Production Through Down-Regulation of NF-κB and Activation of Nrf2-Mediated HO-1 in BV2 Microglial Cells Treated with LPS. Molecules.

[37] Yau K.I., Huang C.B., Chen W., Chen S.J., Chou Y.H., Huang F.Y., Kua K.E., Chen N., McCue M., Alarcon P.A. (2003). Effect of nucleotides on diarrhea and immune responses in healthy term infants in Taiwan. J. Pediatr. Gastroenterol. Nutr..

[38] Xu M., Zhao M., Yang R., Zhang Z., Li Y., Wang J. (2013). Effect of dietary nucleotides on immune function in Balb/C mice. Int. Immunopharmacol..

[39] Morozov Y.M., Datta D., Paspalas C.D., Arnsten A.F.T. (2017). Ultrastructural evidence for impaired mitochondrial fission in the aged rhesus monkey dorsolateral prefrontal cortex. Neurobiol. Aging.

[40] Pollard A.K., Craig E.L., Chakrabarti L. (2016). Mitochondrial Complex 1 Activity Measured by Spectrophotometry Is Reduced across All Brain Regions in Ageing and More Specifically in Neurodegeneration. PLoS ONE.

[41] Jafari A., Hosseinpourfaizi M.A., Houshmand M., Ravasi A.A. (2005). Effect of aerobic exercise training on mtDNA deletion in soleus muscle of trained and untrained Wistar rats. Br. J. Sport. Med..

[42] Arnaud A., López-Pedrosa J.M., Torres M.I., Gil A. (2003). Dietary nucleotides modulate mitochondrial function of intestinal mucosa in weanling rats with chronic diarrhea. J. Pediatr. Gastroenterol. Nutr..

[43] Wu Z., Puigserver P., Andersson U., Zhang C., Adelmant G., Mootha V., Troy A., Cinti S., Lowell B., Scarpulla R.C. (1999). Mechanisms controlling mitochondrial biogenesis and respiration through the thermogenic coactivator PGC-1. Cell.

[44] Herskovits A.Z., Guarente L. (2013). Sirtuin deacetylases in neurodegenerative diseases of aging. Cell Res..

[45] Klimova N., Kristian T. (2019). Multi-targeted Effect of Nicotinamide Mononucleotide on Brain Bioenergetic Metabolism. Neurochem. Res..

[46] Chen X., Amorim J.A., Moustafa G.A., Lee J.J., Yu Z., Ishihara K., Iesato Y., Barbisan P., Ueta T., Togka K.A. (2020). Neuroprotective effects and mechanisms of action of nicotinamide mononucleotide (NMN) in a photoreceptor degenerative model of retinal detachment. Aging.

[47] Zhu N., Liu X., Xu M., Li Y. (2021). Dietary Nucleotides Retard Oxidative Stress-Induced Senescence of Human Umbilical Vein Endothelial Cells. Nutrients.

[48] Pluvinage J.V., Wyss-Coray T. (2020). Systemic factors as mediators of brain homeostasis, ageing and neurodegeneration. Nat. Rev. Neurosci..

[49] Montagne A., Barnes S.R., Sweeney M.D., Halliday M.R., Sagare A.P., Zhao Z., Toga A.W., Jacobs R.E., Liu C.Y., Amezcua L. (2015). Blood-brain barrier breakdown in the aging human hippocampus. Neuron.

